# Emergence of a substrate-temperature-dependent dielectric process in a prototypical vapor deposited hole-transport glass

**DOI:** 10.1038/s41598-018-19604-7

**Published:** 2018-01-22

**Authors:** Cristian Rodríguez-Tinoco, Marzena Rams-Baron, Javier Rodríguez-Viejo, Marian Paluch

**Affiliations:** 1 Institute of Physics, University of Silesia, 75 Pulku Piechoty 1A, 41-500 Chorzow, Poland; 2 Silesian Center for Education and Interdisciplinary Research, 75 Pulku Piechoty 1A, 41-500 Chorzow, Poland; 3 Group of Nanomaterials and Microsystems, Physics Department, Universitat Autònoma de Barcelona, 08193 Bellaterra, Spain

## Abstract

Since the discovery of ultrastability, vapor deposition has emerged as a relevant tool to further understand the nature of glasses. By this route, the density and average orientation of glasses can be tuned by selecting the proper deposition conditions. Dielectric spectroscopy, on the other hand, is a basic technique to study the properties of glasses at a molecular level, probing the dynamics of dipoles or charge carriers. Here, and for the first time, we explore the dielectric behavior of vapor deposited N,N-Diphenyl-N,N’bis(methylphenyl)-1,1′-biphenyl-4,4′-diamines (TPD), a prototypical hole-transport material, prepared at different deposition temperatures. We report the emergence of a new relaxation process which is not present in the ordinary glass. We associate this process to the Maxwell-Wagner polarization observed in heterogeneous systems, and induced by the enhanced mobility of charge carriers in the more ordered vapor deposited glasses. Furthermore, the associated activation energy establishes a clear distinction between two families of glasses, depending on the selected substrate-temperature range. This finding positions dielectric spectroscopy as a unique tool to investigate the structural and electronic properties of charge transport materials and remarks the importance of controlling the deposition conditions, historically forgotten in the preparation of optoelectronic devices.

## Introduction

Glasses are object of an intense research due to its importance in industrial applications. Many drugs used in the pharmaceutical industry are more soluble and easier to stabilize in the amorphous state rather than in some crystal polymorphs^1,2^. The solid-like mechanical properties and the ease of producing large areas of glassy films with macroscopic homogeneity has been exploited by the electronic industry, extensively using glasses in organic electronic or photovoltaic devices^3–5^. These are some examples of the ubiquity of glasses in nature and technology.

There are several routes to produce glasses: fast-quenching^6^, cryo-milling^7^ or high pressure quenching^8^, among others. However, the discovery of ultrastable glasses (USG) by vapor deposition (VD) represents a milestone in glass research^9,10^. By playing with the deposition conditions (mainly deposition temperature, *T*_*dep*_, and evaporation rate), it is possible to tune the properties of the produced glass. In particular, one can prepare molecular glasses with a thermodynamic stability and density ranging along a very wide range^11^, from those which compare to ordinary glasses (OG) to the so-called ultrastable glasses (USG), equivalent to ambers aged for millions of years^12^. Apart from the higher density and stability level, vapor deposited glasses also present a series of striking properties different from those exhibited by ordinary glasses. For example, at sufficiently high temperature, they transform into the super-cooled liquid via a heterogeneous transformation mechanism, instead of the homogeneous transformation process of ordinary glasses^13–15^. Even more exciting is the presence of certain molecular ordering, which has been observed by different experimental techniques^11,16,17^. The control of the molecular orientation in solids is, in fact, one of the long-standing goals of chemistry. Molecular ordering in vapor deposited glasses is believed to have impact also on other properties, including the transformation behavior^18^ or the suppression of the two-level systems at low temperatures^19^. Vapor deposition offers, therefore, a new benchmark to test the theories concerning the nature of glasses, since it permits to cover a wide range of thermodynamic stability, not accessible by other means, and to study the effect of molecular orientation in the properties of molecular glasses. One of the most exciting application of ordering in molecular glasses is the field of organic electronics and, in fact, we can find in the literature several examples where the charge carrier mobility of different materials is improved by the control of structural anisotropy^20–22^. By controlling the orientation of the molecules, it is possible to enhance the overlap in *π* molecular orbitals, improving the charge carrier efficiency. This effect has been extensively studied in polymeric, crystalline and liquid crystals^23–25^. The irruption of vapor deposited glasses has boost this field of research.

The study of dielectric processes is fundamental for the influence of those in the properties of glasses. For example, the origin of secondary relaxations^26^, that remain active in the glass when all structural evolution is frozen, is still not clear^27^. Secondary processes are responsible for several features of glasses^28,29^. Very recently, the very nature of ultrastable vapor deposited glasses was correlated to the timescale of the Johari-Goldstein (JG) secondary relaxation^30^. On the other hand, intermolecular charge transport, which also originates a dielectric response in the material, is a central concern of fundamental and practical nature for optoelectronic or energy applications. As an example, protic ionic liquids, of crucial importance in a wide range of emerging technologies, have long been envisioned as promising candidates for electrolytes in fuel cells, green solvents or batteries. The study of their dielectric properties by means of broadband dielectric spectroscopy (BDS) has become a very active field^31^. BDS has been also widely used to measure conductive properties of disordered organic semiconductor systems^32–35^, proving to be a very powerful technique to understand the underlying mechanism of charge carrier transport in them, essential to improve the device performance. Impedance spectroscopy, similar to BDS, has been used to obtain, from the frequency dependent capacity, charge carrier mobilities, geometric capacitance or built-in potential in some devices^36^.

In spite of the unconventional and intriguing nature of vapor deposited glasses, little research has been carried out concerning the dielectric processes on them. Recently, it was shown that the secondary relaxation processes in USG of toluene^37^ and etoricoxib^38^ is slightly slower and exhibit much lower intensity, and its properties are linked to those of the structural relaxation^38^. However, while this effect is attributed to the much higher density of USG compared to the ordinary counterpart, there is no work considering the possible effect of molecular orientation in the dielectric response of vapor deposited glasses. Molecular orientation is especially important in the performance of organic electronic devices^39,40^. To address this point, we propose to study the dielectric behavior of vapor deposited glasses of N,N-Diphenyl-N,N′ bis(methylphenyl)-1,1′-biphenyl-4,4′-diamines (TPD) (*T*_*g*_ = 330 *K*), a prototypical organic hole-transport molecule widely used in electronic applications, deposited at different substrate temperatures, covering a wide range of density values and average molecular orientation. TPD and its derivatives have been widely studied as model hole transport material and are used in several industrial applications such as photocopying devices, white light electroluminescent devices, voltage-tunable color organic light-emitting diodes (LEDs) or low cost flat panel displays^41^.

## Results and Discussion

**Figure 1:**
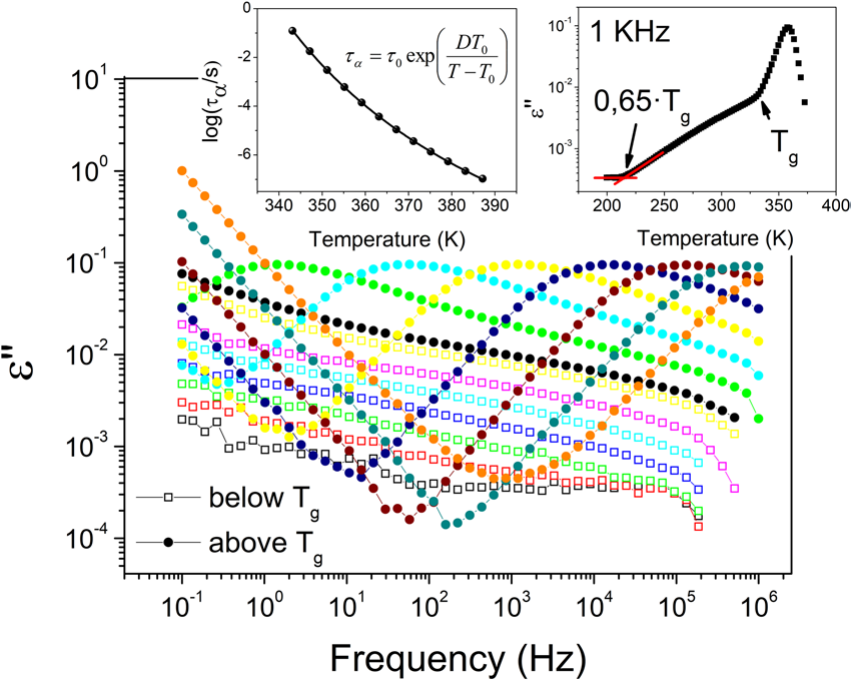
Permittivity of TPD samples below (open symbols) and above (closed symbols) the glass transition temperature. Spectra in the glassy state correspond to T = 213 K–333 K, with ${\rm{\Delta }}T=20K$. Spectra in the liquid state correspond to T = 335 K–391 K, with ${\rm{\Delta }}T=8K$. In the left inset, relaxation time of TPD super-cooled liquid, fitted with the shown VFT equation, using the following parameters: $\mathrm{log}({\tau }_{0})=-16.41$, $D=8.82$ and ${T}_{0}=275.23$. In the right inset, permittivity of TPD at a fixed frequency (1 kHz) as a function of temperature, showing NCL-like behavior.

In Fig. 1 we show the dielectric loss spectra of TPD in the glassy state (below the glass transition temperature, *T*_*g*_) and in the liquid state (above *T*_*g*_). While we observe the structural relaxation in the liquid state, the spectra corresponding to the glassy state are featureless. In fact, from the slope of the dielectric loss spectra of ordinary TPD, we can evidence that it corresponds to a nearly constant loss (NCL), which is attributed to the dissipation of molecular movements confined by an anharmonic potential (cage dynamics)^42^. NCL is usually observed at very high frequencies by THz spectroscopy or in a relatively small range of frequencies in polymers or small glass formers^43^. However, due the lack of a secondary relaxation peak in TPD OG, it can be observed at lower frequencies. By plotting the intensity of the NCL versus temperature, we observe a change in slope at around 0.65*T*_*g*_ (see right inset in Fig. 1). Very recently, the same phenomena were observed in 1,6-anhydro-D-glucose and 1,6:2,3-dianhydro-b-D-mannopyranose^42^. A similar behavior has been reported by Sibik *et al*. for polyalcohols^44^ and discussed in a series of papers by Capaccioli *et al*. as connected to the freezing of the Johari-Goldstein secondary relaxation process^43,45,46^. In fact, according to reported results^17^, below *T*_*dep*_ ≈ 0.65*T*_*g*_, vapor deposited glasses are no longer denser than the ordinary glass, consistent with the association between JG relaxation and formation of VD glasses with enhanced density^30^. The presence of the JG process is perceived from the particularly broad aspect of the structural relaxation process in the high-frequency flank, which seems to indicate the presence of the often-discussed excess wing, a typical feature studied in other glassy systems and associated to a hidden JG relaxation^47,48^.

**Figure 2:**
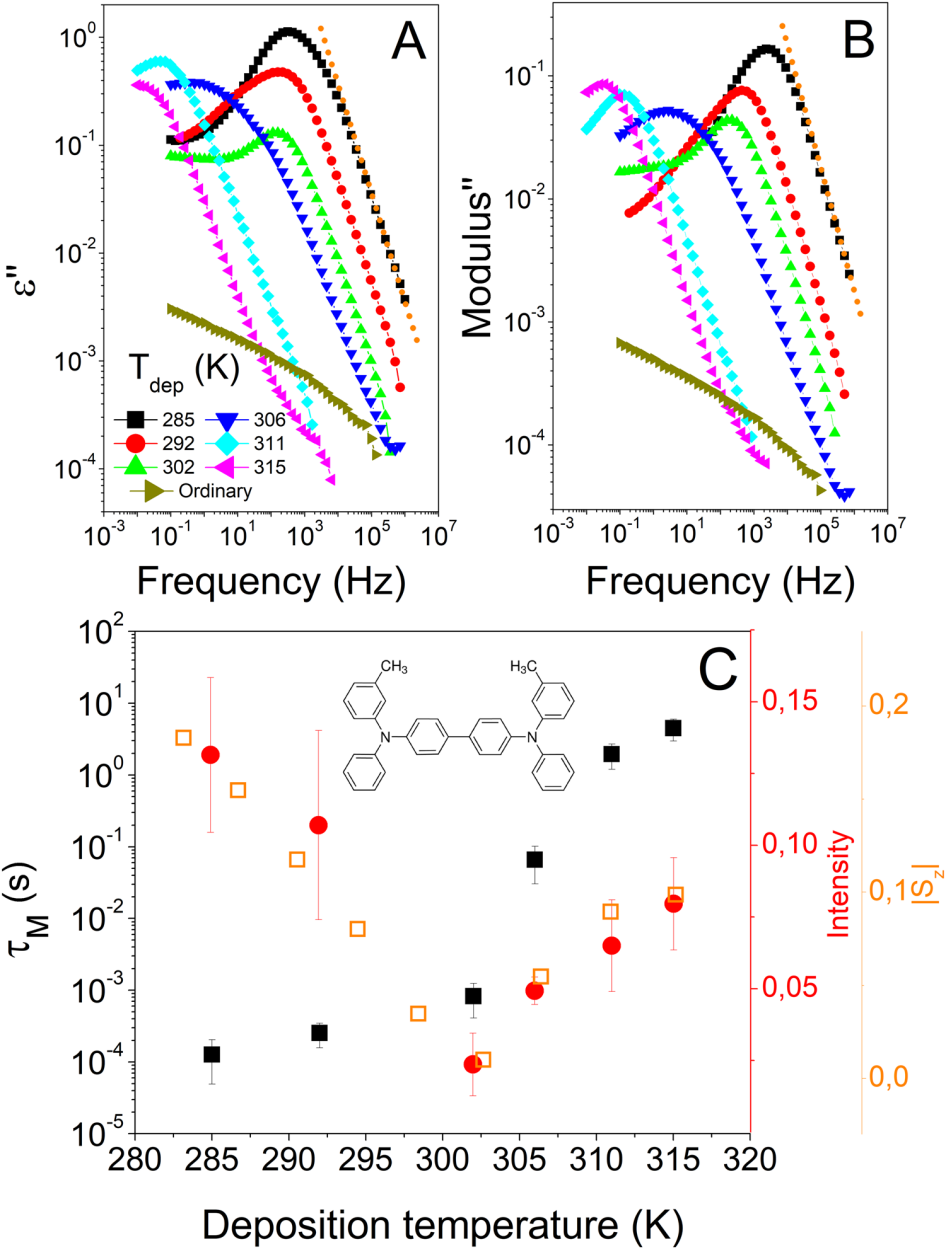
Dielectric loss of TPD glasses produced at different conditions in permittivity (**A**) and modulus representation (**B**), measured at 283 K. The orange dotted line represented the function $\Phi (f)=A{f}^{-1}$. We only include one spectra for each deposition temperature, representative of the average behavior. The complete set of measured samples can be found in the supplementary information. (**C**) Relaxation time (left axis) and peak intensity (inner right axis, red filled symbols) of the dielectric process as a function of deposition temperature, obtained from the modulus representation. We also plot the absolute value of the order parameter calculated by Dalal *et al*.^17^ (outer right axis, orange open symbols). Error bars in time and intensity have been obtained from the standard deviation of the average from measurement in different equivalent samples. A representation of the TPD molecule is also included.

In Fig. 2, we show both the dielectric modulus and the dielectric permittivity spectra of TPD vapor deposited glasses produced at different substrate temperatures and measured at 283 *K*. In clear contrast with the ordinary glass, the permittivity (left panel) and modulus (right panel) of vapor deposited glasses clearly exhibit a pronounced dielectric peak. To discard any artefact during the dielectric measurement, we have transformed a VD glass on a thermal plate prior to the measurement and then measured following the same protocol as with the as-deposited samples. Alternatively, we have also prepared an ordinary glass sample of TPD on a thermal plate and then grinded and placed it on top of a BDS electrode (as seen in Methods). The output from these two described cases are the same as the one reported under the label of ordinary. The intensity and the position (the latter related to the relaxation time) of this process depend on the deposition conditions, as represented in Fig. 2C. Strikingly, the higher the density of the VD glass, according to the reported values^17^, the higher the frequency of the dielectric process (at *T*_*dep*_ = 285 *K* ∼ *0.86T*_*g*_ density is the highest from the shown VD samples, while at *T*_*dep*_ = 315 *K* ∼ *0.95T*_*g*_ the density is the lowest). This result is unexpected, since, according to previous reports on secondary relaxations, one would expect slower or unaffected relaxation times for denser systems^8,37,49^. Interestingly, at frequencies above the peak maximum, the logarithm of the dielectric loss is inversely proportional to the logarithm of frequency (dotted line in Fig. 2A and B). This is a strong evidence that the process is not originated from dipolar relaxation, but is the Maxwell-Wagner (MW) process, characterized by Debye-type relaxation behavior^50^. MW processes are related to conductivity heterogeneities and charge transfer phenomena. We will come back to this point later. We note that in a permittivity representation the dispersion of results is much higher (see Figures S1 and S2 in the supplementary information), which also points in the direction of a conductivity-related effect.

**Figure 3:**
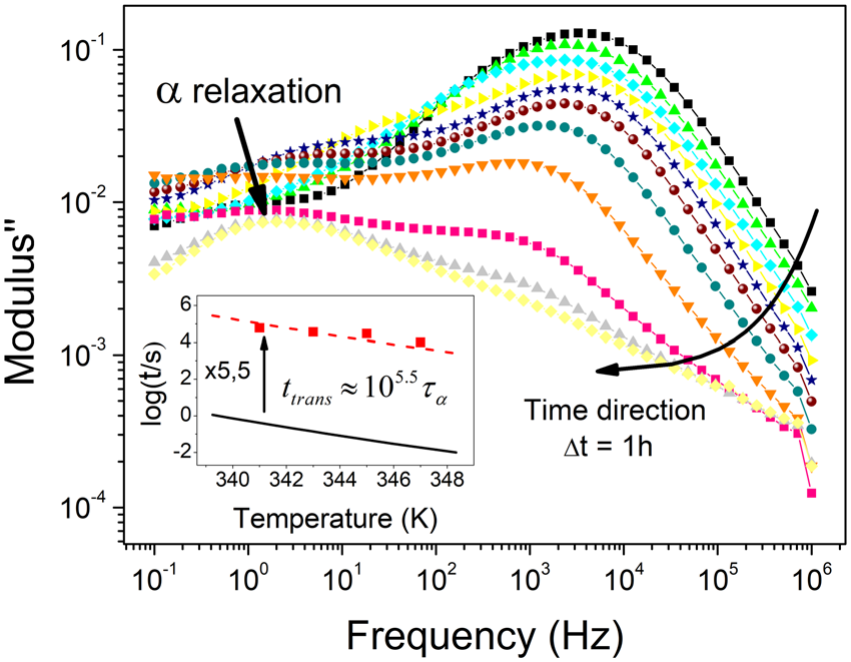
Transformation of an ultrastable TPD glass (T_dep_ = 285 K ∼ 0.86T_g_) at 343 K (T_g_ + 13 K). Each curve is obtained after one hour at the annealing temperature. It can be observed the emergence of a second process that shift towards lower frequency values. In the inset, transformation time of ultrastable TPD at different temperatures, together with the VFT function corresponding to super-cooled TPD, showing that the transformation time of ultrastable TPD is approximately 5.5 orders of magnitude larger than the relaxation time of the super-cooled liquid.

To clearly illustrate the idea of an emerging process we analyze the evolution of the dielectric spectra of an USG (*T*_*dep*_ = 285 *K* ∼ *0.86T*_*g*_) at 343 *K* (*T*_*g*_ + 13 *K*), represented in Fig. 3. The prominent initial dielectric process gradually decreases in intensity as time evolves. At certain moment, the structural relaxation of the liquid starts to be appreciated. When the sample is completely transformed, the initial high intensity peak has completely vanished. This observation clearly points out that the exhibited process is intrinsically related to the glassy state. Due to the high density and thermodynamic stability of the USG, the transformation from glass to liquid above *T*_*g*_ takes several orders of magnitude longer than the structural relaxation time of the liquid at that temperature. We observe this trend in the inset of Fig. 3, where we show that the transformation time of TPD USG at different temperatures is approximately 5.5 orders of magnitude higher than the corresponding structural relaxation time of the liquid. This fact was already reported for TPD as well as for many other molecular glass-formers^51–53^. We note that the Debye-like aspect of the process is preserved until the latest stages of the transformation. Only at advanced stages of the transformation process, the position of the main peak is significantly shifted and the Debye-like aspect is lost (pink squares in Fig. 3).

**Figure 4:**
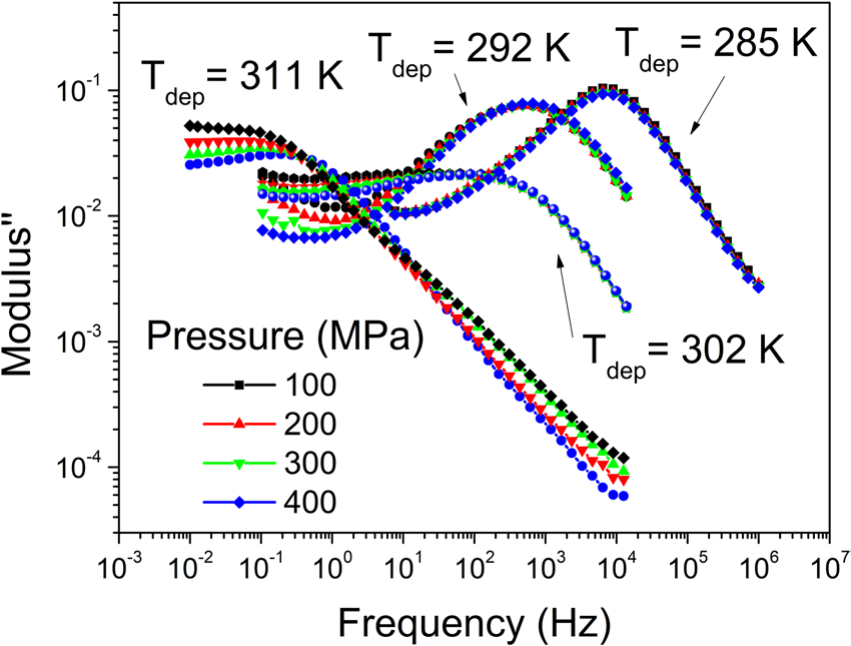
Electric modulus of TPD glasses vapor deposited at different deposition temperatures, measured at 293 K and at variable pressure (as indicated in the legend).

In Fig. 4 we show the dielectric spectra of VD glasses at elevated pressure. For the USG (*T*_*dep*_ = 285 *K* ∼ *0.86T*_*g*_), there is no significant change of peak position with pressure, indicating that it is not significantly affected by the density. In the case of a less stable glass (*T*_*dep*_ = 311 *K* ∼ *0.94T*_*g*_) the applied elevated pressure slightly shifts the process towards higher frequency values. That the peak frequency position from the glass grown at *T*_*dep*_ = 311 *K* ∼ *0.94T*_*g*_ increases with pressure reinforces the assumption that the origin of the process is not a dipolar relaxation, since we do not expect that a homogenous increase in density would yield a faster dipolar relaxation process. Also, that an increase in density induced by an increase in pressure does not significantly alter the position of the dielectric process in the denser TPD glasses is an indication that the process is independent of glass density.

Vapor deposited glasses present two main characteristics: the possibility of exhibiting high values of density and a special molecular arrangement. These two properties are substrate temperature dependent. From the invariance of peak position under elevated pressure (Fig. 4), we rule out a determining influence of density in the process. However, glasses grown at different *T*_*dep*_, and hence with different density, exhibit very different dielectric processes (Fig. 2). Therefore, we may assume that the observed emergent relaxation process in the glassy state originates from the particular molecular orientation of vapor deposited glasses. According to Dalal *et al*.^17^, the average molecular orientation of VD glasses of TPD strongly depends on the deposition conditions. Glasses deposited below approximately 302 *K* ∼ *0.92T*_*g*_ exhibit a negative ellipsometry derived order parameter (*S*_*z*_ < 0), meaning that the long axis of TPD molecules has a tendency to align parallel to the substrate, being the tendency stronger as the deposition temperature is reduced. Further experiments with WAXS show that the molecules have a tendency to lie in face-on configuration, i.e. the central phenyl rings from each molecule are facing each other. On the other hand, glasses deposited above 302 *K* ∼ *0.92T*_*g*_ present a positive value of *S*_*z*_ (*S*_*z*_ > 0), consistent with molecules with a tendency to orient with the long axis perpendicular to the substrate. In that range, there is a maximum in *S*_*z*_ at around *T*_*dep*_ = 315 *K* ∼ *0.95T*_*g*_. The anisotropy in VD glasses is considered to be a direct consequence of molecular orientation at the surface of thin films of equilibrium liquid. At the very surface, molecules in an equilibrated liquid are prone to align parallel to the surface. In an intermediate layer, molecules are prone to align perpendicular to the substrate. Further from the surface, in the bulk, molecules are already isotopically oriented. As *T*_*dep*_ increases, molecules landing onto the substrate during the evaporation process have more time to align throughout a deeper position in the thin layer, yielding the differences reported in molecular orientation in VD glasses as a function of *T*_*dep*_^17^. In Fig. 2 C we plot the intensity of the dielectric process as a function of *T*_*dep*_, together with the reported values of the absolute value of the average orientation. According to that figure, the higher the degree of orientation in the sample (regardless of the direction of the orientation), the higher the intensity of the dielectric process. This observation reinforces the idea that molecular orientation induces the emergence of the new process. We note, however, that the frequency position of the peak does not correlate with orientation. Since the relative position of the molecules with respect to the electric field applied during the BDS measurement is different depending on the sample, we would not expect such correlation to take place. In other words, while molecular orientation induces a dielectric process, regardless of the particular direction, the dynamic response of that process does depend on the direction.

**Figure 5:**
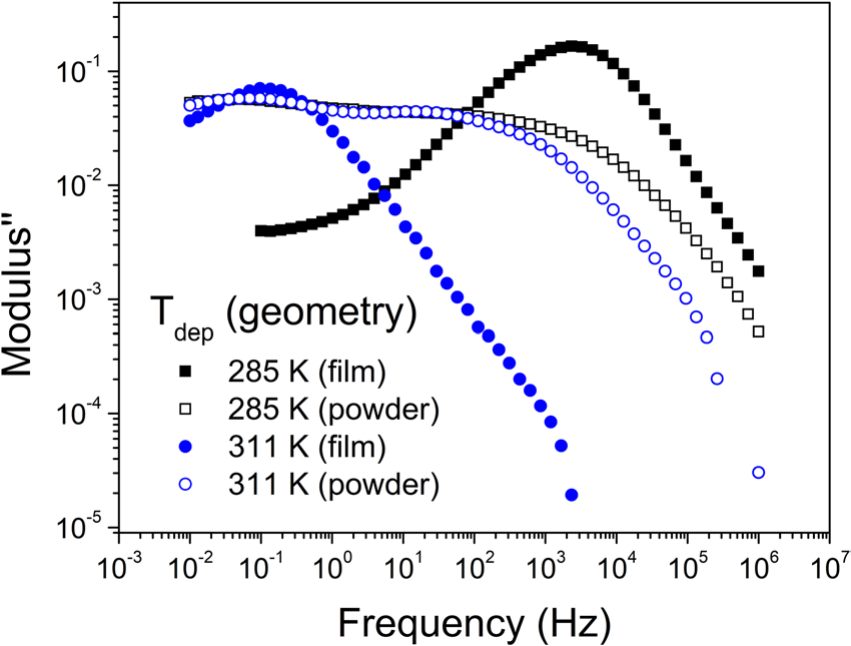
TPD glasses deposited at 285 K ∼ 0.86T_g_ (black squares) and 311 K ∼ 0.94T_g_ (blue circles) in thin film geometry deposited directly on top of the capacitor (closed symbols) and scratched from an aluminum substrate and placed on top of the capacitor (open symbols).

To further verify this point, we compare the spectra obtained from a sample in thin-film configuration (as-deposited) with a sample grown at the same conditions but after being scratched from the substrate and converted to powder. If the observed dielectric process is originated by the average orientation of the molecules, then a change in the macroscopic orientation of the sample with respect to the electric field applied during the measurement should result in a change in the dielectric spectra. The results are plotted in Fig. 5 and confirm our assumption. While the film grown at 311 *K* ∼ *0.94T*_*g*_ exhibits a considerably narrow dielectric peak, the same sample in powder configuration present, in addition, some contribution to the dielectric spectra at higher frequencies. When the sample is scratched, the resulting pieces become randomly oriented and this give rise to a dielectric contribution from molecules oriented parallel to the substrate. An analogous behavior is observed for a glass grown at 285 *K* ∼ *0.86T*_*g*_. We note that the onset of devitrification of the glass in film or powder configuration remains the same, indicating that the thermodynamic or kinetic state of the glass is unaffected by the scratching process (see Figure S3 in the supplementary information).

**Figure 6:**
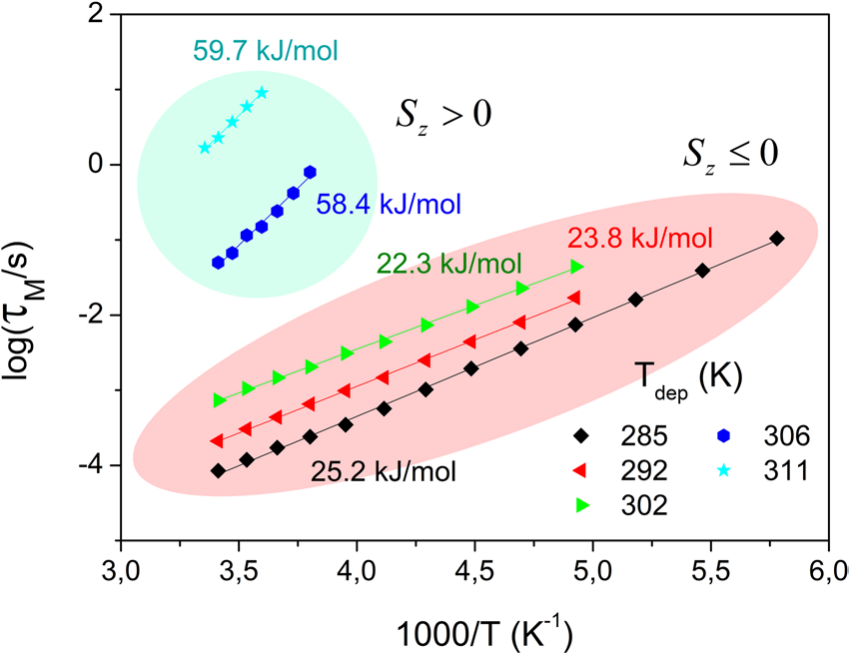
Logarithm of the relaxation time (from modulus representation) in glasses deposited at different temperatures as a function of (1000/T). The shaded regions indicate glasses with positive order parameter (green) and negligible or negative order parameter (red), according to reported data^17^.

In Fig. 6 we represent the Arrhenius plot of the relaxation time (in modulus representation) associated with the emerging dielectric process. It is surprising that, on one hand, glasses with *S*_*z*_ ≤ 0 (*T*_*dep*_ = 285 *K* ∼ *0.86T*_*g*_, 292 *K* ∼ *0.88T*_*g*_ and 302 *K* ∼ *0.92T*_*g*_) have a very similar value of the activation energy, even though the average orientation in those glasses significantly changes (for *T*_*dep*_ = 302 *K* ∼ *0.92T*_*g*_, *S*_*z*_ is approximately 0). The same is observed for glasses with *S*_*z*_ > 0 (*T*_*dep*_ = 306 *K* ∼ *0.93T*_*g*_, 311 *K* ∼ *0.94T*_*g*_). On the other hand, glasses with similar average orientation (292 *K* ∼ *0.88T*_*g*_ and 311 *K* ∼ *0.94T*_*g*_) but oriented along different directions exhibit a very different value of activation energy. Interestingly, by considering the activation energy of the observed process, a clear distinction emerges between the two families of glasses, those with positive and those with negative order parameter, i.e. glasses where, on average, molecules lie on top of the surface or are perpendicular to it (face-on or edge-on, respectively).

**Figure 7:**
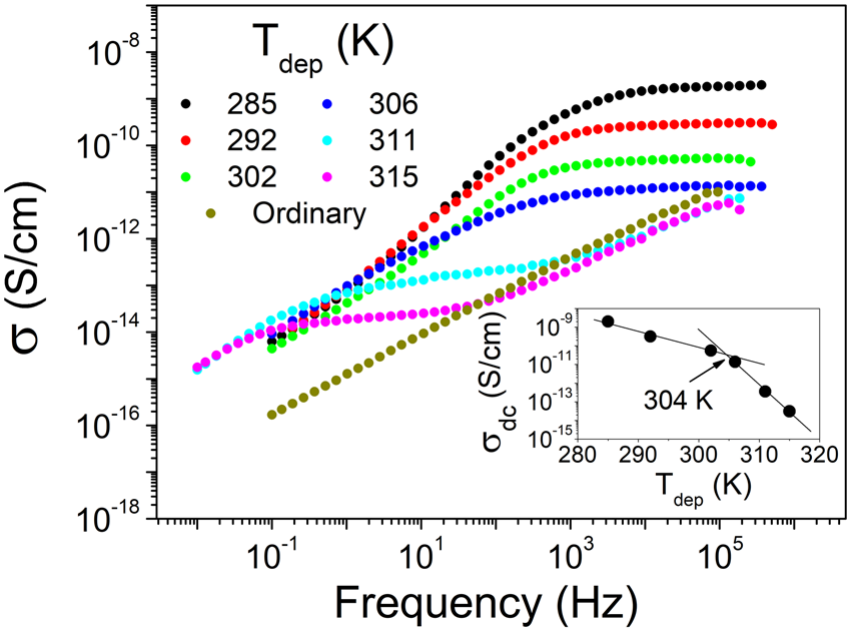
AC conductivity of TPD glasses deposited at different substrate temperatures. In the inset, the DC conductivity of each glass at the frequency value that yields the maximum in the modulus spectra (Fig. 2), corresponding to the lowest frequency value in the plateau region in the AC curves.

If we assume that molecular orientation induces the emergence of a process that is blocked for the completely isotropic ordinary glass, the question is, what is the underlying physical mechanism? As observed from Fig. 2, the high frequency side of the emerging dielectric process is directly proportional to the logarithm of the frequency with slope -1, characteristic of Debye processes. As we have seen, the characteristics of the process are unaltered through changes in density by pressurization. From all these observations, we conclude that the dielectric process is the Maxwell-Wagner polarization. This process is typical of inhomogeneous materials, where regions with different electrical conductivity induce the formation of interfacial charge accumulations and, as a consequence, the emergence of an equivalent dipolar moment with characteristics of a Debye relaxation process. The presence of a MW process implies, therefore, heterogeneous structure and electrical conductivity. That molecular orientation enhances charge transfer between molecules is a well-known fact^39,40^. Yokoyama *et al*. demonstrated for BSB-Cz that the orientation anisotropy of VD glasses, which depends on deposition conditions, can improve their charge carrier transport characteristics with respect to the ordinary isotropic glass^20^. The same effect was also recently observed in computational simulations of ethylbenzene^54^. The site energy of the hole state of a molecule depends strongly on the relative orientation of the adjacent dipoles^55^. According to some theories, in the case of more structurally ordered systems, the dipolar environment of each molecule is more uniform and that leads to a reduced energetic disorder and, consequently, to an increase in the charge transport efficiency^39^. From another point of view, a stronger interaction of delocalized electrons through increased *π-π* stacking yield a higher conduction efficiency. We note that, according to the reported results, there is no charge conduction from electrode to electrode, and consequently no charge injection into the system. Even though the applied voltage is considerably low (1 *V*, or *E* ∼ 3·10^4^ *V/m*), a small amount of charge carriers may exist due to temperature effects. The low values of AC conductivity, shown in Fig. 7, support this fact. We note also from these data the considerable increment of DC conductivity (plateau in the conductivity spectra) in VD glasses as *T*_*dep*_ becomes lower, at frequencies corresponding to the MW process (inset of Fig. 7). Higher electrical conductivity yields a faster MW process.

**Figure 8:**
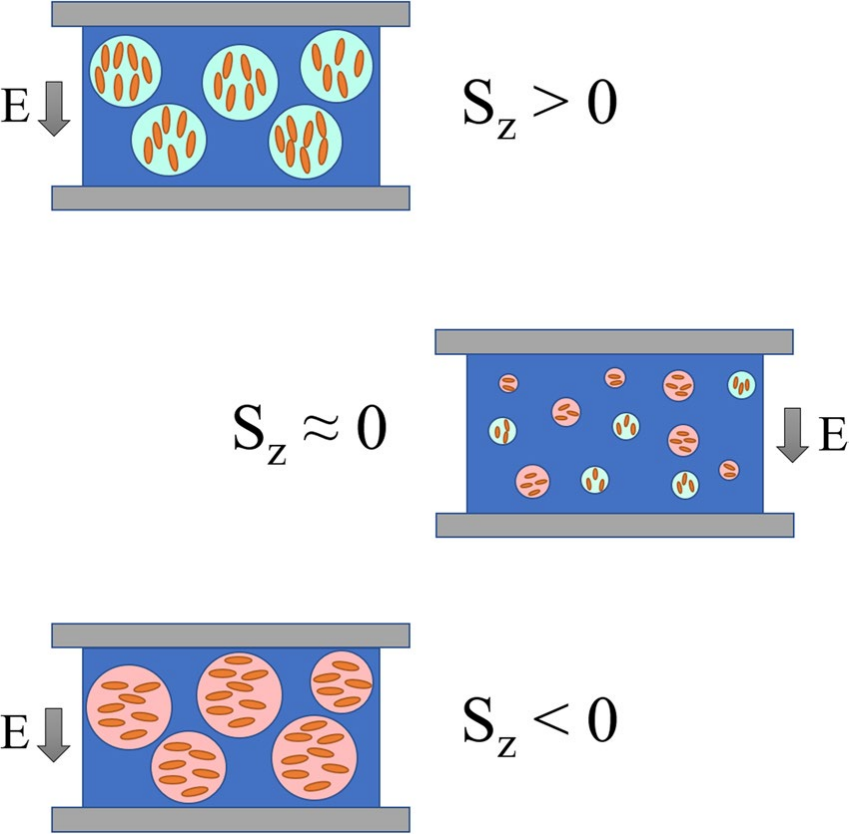
Schematic view of the structure of VD glasses of TPD at different T_dep_, according to the interpretation of the presented results. Clusters where molecules have a tendency to align along a particular direction are surrounded by regions where molecular orientation is slightly different (and therefore, conductivity is also different) or by completely isotropic regions. Depending on T_dep_, the orientation inside the clusters varies. In the case of glasses deposited near 302 K ∼ 0.92T_g_, regions with opposite molecular orientation coexist, yielding a negligible average orientation but, still, exhibiting different low intensity MW polarizations.

Under this framework, we interpret the VD glasses as composed of clusters where molecules have a slight tendency to be oriented along a particular direction. The higher the degree of on-plane orientation (perpendicular to the applied field) in these clusters, the higher the electrical conductivity and the faster the originated MW process. The association between these clusters and the cooperative rearranging regions (CRR) considered in glass science^56^ is an appealing open issue, and it seems reasonable that, inside a particular CRR, molecules will tend to align in a similar way, while molecules in a different CRR may be able to align with a different angle. It is also surprising that glasses deposited at *T*_*dep*_ = 302 *K* ∼ *0.92T*_*g*_, with *S*_*z*_ *≈* 0 according to reported values present also MW polarization with activation energy similar to the glasses deposited at higher temperatures. Moreover, after closer inspection of the spectra corresponding to the glass deposited at 306 *K* ∼ *0.93T*_*g*_, also with an almost negligible order parameter, we can see that it exhibits a small contribution from a fast process, at high frequencies (similar to where we observe the process of the glass at *T*_*dep*_ = 302 *K* ∼ *0.92T*_*g*_), together with the main contribution at lower frequencies (a clearer illustration of this is given in Figure S4 in the supplementary information). We argue that, due to temperature or intrinsic heterogeneities in the glassy structure, glasses with negligible average order parameter exhibit small regions where molecules are slightly oriented parallel to the substrate, coexisting with regions where molecules are slightly oriented perpendicular to the substrate. This interpretation is represented in Fig. 8.

Finally, the relationship between DC conductivity values and *T*_*dep*_ shown in the inset of Fig. 7 exhibits a kink at ∼ 304 K, approximately the *T*_*dep*_ at which the overall structure is isotropic. The same kink is appreciated in the representation of the relaxation time associated with the MW process as a function of *T*_*dep*_ observed in Fig. 2C, which is a consequence of the link between the process and the conductivity of the sample. In Fig. 6 we saw that the activation energy of the MW process also changes at approximately the same *T*_*dep*_. All these observations suggest that there is a point of inflection in the characteristics of the process at ∼302 K, reported as the temperature at which *S*_*z*_ = 0. Various explanations are at hand to try to rationalize this finding. For example, depositions at above or below 302 *K* may yield glasses with different structure, even if sharing the same absolute value of *S*_*z*_. In this sense, the local environment of molecules influences the charge transport properties, and does not necessary have to follow the same trend with *T*_*dep*_ as the average molecular orientation. Alternatively, even though density is not responsible for the emergence of the process, it may play a role in the characteristics of glasses deposited above 302 *K*. In this sense, it is also interesting to note, from Fig. 4, that changes in density induced by pressurization seems to affect the dielectric spectra at higher frequencies, i.e., while the glass grown at *T*_*dep*_ = 285 *K* ∼ *0.86T*_*g*_ is not affected by pressure, the glass grown at *T*_*dep*_ = 311 *K* ∼ *0.94T*_*g*_ exhibits a faster process upon pressurization. In any case, the answer to this issue is far from trivial and further work is required to give a complete answer.

## Conclusions

We report the emergence of the Maxwell-Wagner dielectric process in the glassy state of vapor deposited TPD, which is not observed when the glass is produced by cooling from the super-cooled liquid state. This process exhibits several features that point in the direction of an order-induced dielectric process, i) high pressures hardly affect the relaxation time of the observed process, and ii) the geometry of the sample (whether it is in thin-film or powder configuration) affects the dielectric response of the system. We attribute the emergence of the MW process to two facts: i) the charge carrier transport between molecules, which is enhanced when the molecules are in face-on orientation, with the long molecular axis lying on the surface of the substrate, and ii) the formation of a heterogeneous structure, formed by clusters of slightly oriented molecules. From the dielectric spectra, we have extracted values of carrier conductivity. According to our results, the DC conductivity of TPD glasses can vary several orders of magnitude depending on *T*_*dep*_, which stress the importance of considering this parameter, historically forgotten, when preparing organic glasses for industrial applications. This finding may also represent the emergence of dielectric spectroscopy, a technique specially well suited to measure the properties of other conductive systems, as a new tool to study the properties of charge transfer materials in VD glasses. Furthermore, we suggest that it is possible to extract information regarding the glassy structure from the behavior of the process with respect to temperature in the glassy state, as well as the structural evolution of the system during transformation into the liquid state. While other techniques measuring molecular orientation are able to give average values, we show that dielectric spectroscopy is able to differentiate between different regions inside the macroscopic glassy structure. Further work regarding this point is currently in progress. Altogether, it represents a new research framework with a huge potential to give new insights into the properties of vapor deposited glasses with tunable molecular orientation.

## Methods

### Sample Preparation

Crystalline N,N-Diphenyl-N,N’bis(methylphenyl)-1,1′-biphenyl-4,4′-diamines (TPD) powder with purity higher than 98% was purchased from TCI and used without further purification. 30 *μm* TPD films were grown by thermal evaporation within an ultra-high vacuum (UHV) setup with base pressure of 5 × 10^−8^ mbar. The growth rate was fixed to 0.25 ± 0.02 *nm/s* and measured with a quartz crystal from Inficon. The deposition temperature was controlled using a PID controlled thermal socket. A liquid nitrogen cold trap is used to improve the vacuum quality. The films were grown onto steel electrodes used for Broadband Dielectric Spectroscopy (BDS) and in aluminum foil (see BDS section). The grown samples were removed from the UHV chamber and immediately stored in vacuum bags to reduce to minimum level any possible water interaction. The samples were then removed from the vacuum bags prior to the measurement. Throughout all the process (from production to measurement) samples are exposed to ambient air only for few minutes. However, since water absorption is a delicate issue when dealing with organic samples, we have included in the supplementary information a brief discussion concerning the unlikeliness of any significant effect from water absorption.

Ordinary glasses of TPD were prepared by melting crystalline TPD directly onto a steel electrode on a hot plate at the melting temperature (*T*_*m*_ = 444 *K*), covered by another electrode (separated by Teflon spacers with 0.1 mm of thickness) and then cooled down in a refrigerated cooper plate. Alternatively, an ordinary glass of TPD was prepared on top of aluminum foil on a hot plate at *T*_*m*_ = 444 *K* and then cooled down in a refrigerated copper plate. Subsequently, the glass was scratched from the aluminum foil in form of powder and then placed between two steel electrodes separated by Teflon spacers (0.1 mm thickness).

### Broadband Dielectric Spectroscopy measurements

Isobaric dielectric measurements at atmospheric pressure were performed using a Novocontrol GMBH Alfa analyzer with frequency range from 10^−2^ *Hz* to 10^6^ *Hz*. The measurement temperature was controlled by Quatro temperature controller using a nitrogen gas cryostat with accuracy better than 0.1 *K*. During the measurements, the samples were placed between steel electrodes of the capacitor (15 mm diameter).

Dielectric measurements at elevated pressure were performed using an automatic high-pressure system (Unipress). The sample, out of contact with compression medium by means of a Teflon casing, was fixed to pressure chamber filled with silicon oil. The temperature during the measurement was controlled by means of a closed-circuit cooler connected to the pressure chamber.

Values of relaxation time were calculated from the maximum of the dielectric peaks in modulus representation, using the expression $\tau ={(2\pi {f}_{max})}^{-1}$.

## Electronic supplementary material

Supplementary Information
