# Supplementary material for: Emergence of a substrate-temperature-dependent dielectric process in a prototypical vapor deposited hole-transport glass
Source: Sci Rep. 2018 Jan 22;8:1380. doi: 10.1038/s41598-018-19604-7 (PMC5778027; doi:10.1038/s41598-018-19604-7)
Supplement: Supplementary file 1 — Supplementary Information [file 41598_2018_19604_MOESM1_ESM.pdf]

SUPPLEMENTARY INFORMATION

Emergence of a substrate-temperature-dependent dielectric process in a prototypical vapor deposited hole-transport glass

Cristian Rodríguez-Tinoco[1,2*], Marzena Rams-Baron[1,2], Javier Rodríguez-Viejo[3], Marian Paluch[1,2]

[1]*Institute of Physics, University of Silesia, Uniwersytecka 4, 40-007 Katowice, Poland.*
[2]*Silesian Center for Education and Interdisciplinary Research, 75 Pulku Piechoty 1A, 41-500 Chorzow, Poland.*
[3]*Group of Nanomaterials and Microsystems, Physics Department, Universitat Autònoma de Barcelona, 08193 Bellaterra, Spain.*

*Corresponding author: Dr. Cristian Rodríguez-Tinoco. Institute of Physics. University of Silesia. Uniwersytecka 4, 40-007 Katowice. Poland. E-mail address: cristian.rodriguez-tinoco@smcebi.edu.pl.

Dielectric curves in modulus and permittivity representation

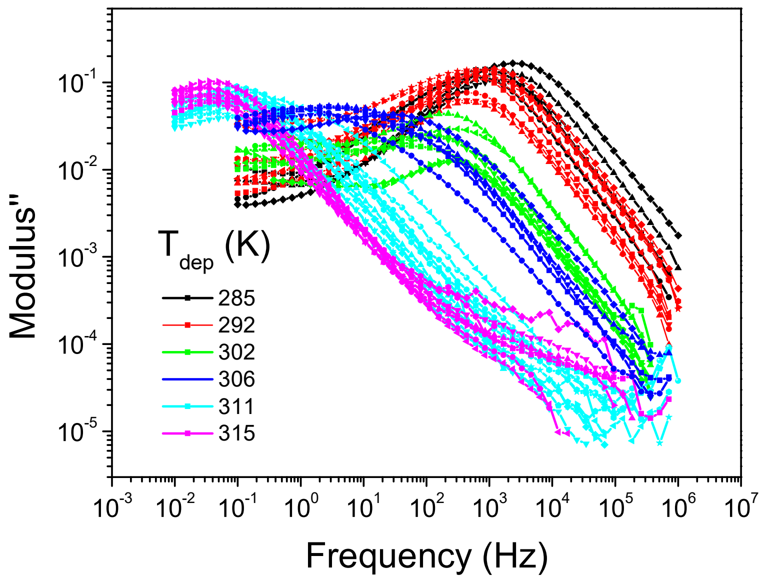

Figure S1. Imaginary part of modulus of several TPD glasses deposited at different substrate temperatures and measured at 283 K. Each color corresponds to different samples growth at the same conditions.

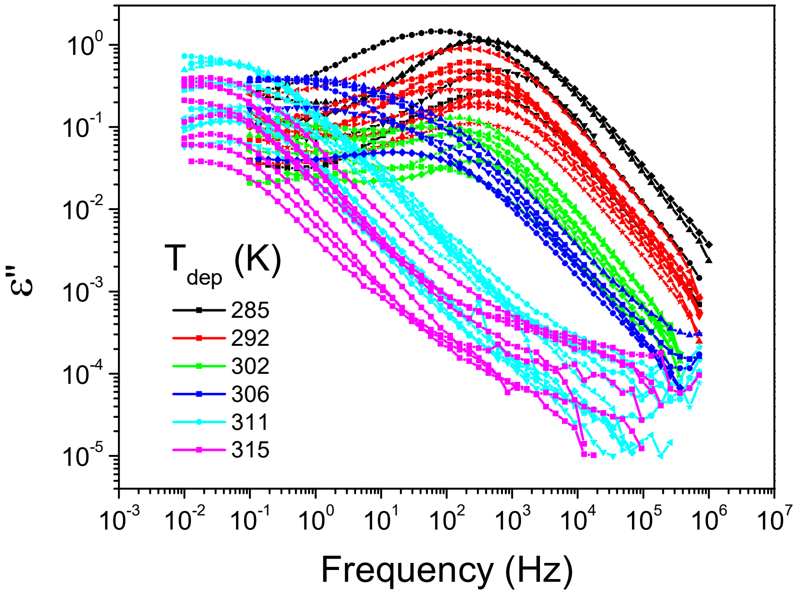

Figure S2. Dielectric loss of several TPD glasses deposited at different substrate temperatures and measured at 283 K. Each color corresponds to different samples growth at the same conditions.

Comparison of as-deposited and scratched samples

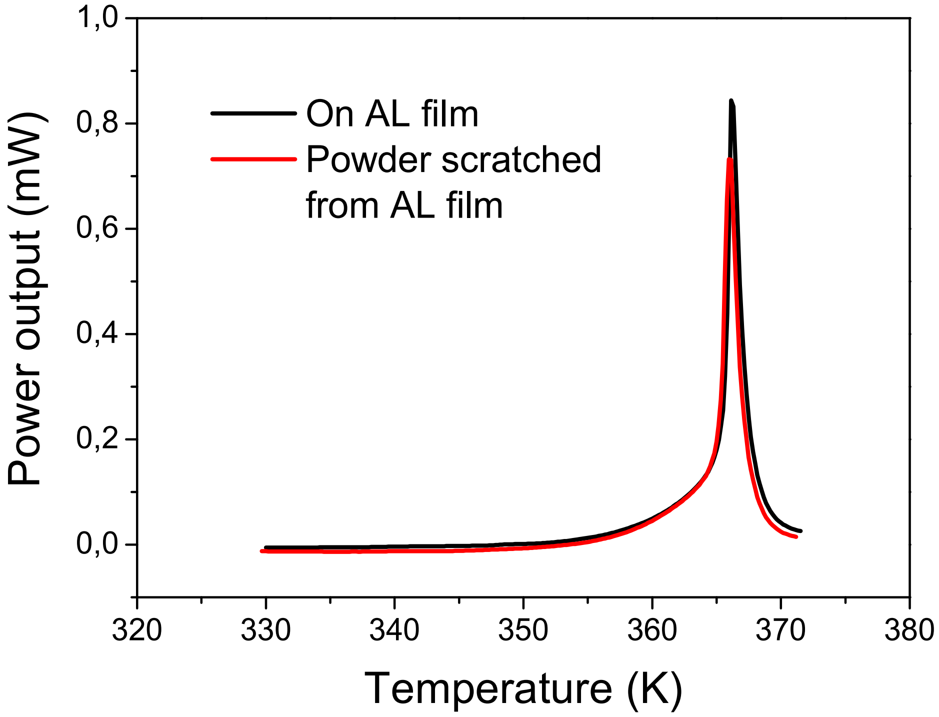

Figure S3. Comparison of the calorimetric trace of two samples grown at the same conditions (T_{dep} = 285 K), where one sample is measured on top of aluminium foil (as-deposited) and the other is scratched from the aluminium foil before measurement. The two curves are practically coincident, indicating that the scratching process does not affect the kinetic stability of the glass.

Coexistence of two peaks in VD glass grown at T_{dep} = 306 K

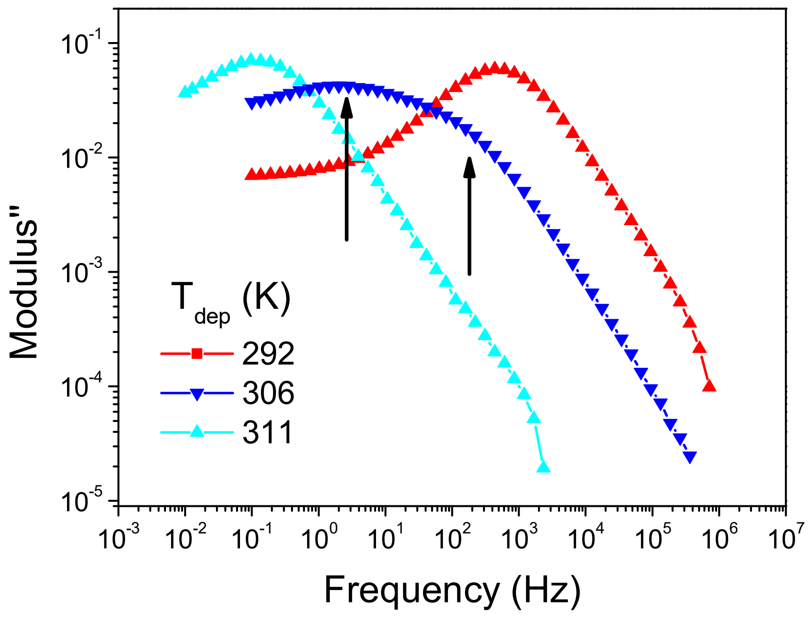

Figure S4. Modulus'' spectra of a glass deposited at 306 K, with order parameter close to 0, remarking the presence of two contributions in the relaxation process. We also show the spectra of a glass deposited at 292 K (negative order parameter) and 311 K (positive order parameter) to compare.

About possible effect of water absorbed in the sample

The grown samples were removed from the UHV chamber and immediately stored in vacuum bags to reduce to minimum level any possible water interaction. The samples were then removed from the vacuum bags prior to the measurement. Throughout all the process (from production to measurement) samples are exposed to ambient air only for few minutes. However, since water absorption is a delicate issue when dealing with organic samples, we provide several reasons to suggest that water is not affecting the characteristics of the measured samples:

- TPD is not hydrophilic and we do not expect water absorption. The same applies to most of the molecules used in the large-scale electronic industry. Also, the

structure is not porous, as in many reported works dealing with the effect of water in organic systems.

- Typically, absorbed water in organic glasses (pharmaceutically glasses, mainly, which are, in most of the cases, highly hydrophilic) acts as a plasticizer, decreasing the associated relaxation time. In a calorimetric scan, absorbed water is reflected with a decrease of the onset of devitrification, with the widening of the transformation peak or, in some cases, with the appearance of additional transformation peaks in the calorimetric scan (related to different areas in the glass structure, depending on their water content). In the case of indomethacin, a pharmaceutical glass former, it was reported that each 1% of water induced a reduction of T_{on} of around 10 K (*Journal of Pharmaceutical Sciences, 86 (3) 346-351 (1997)*). In our case, the calorimetric scans corresponding to the analysed samples do not show any of those easily identifiable features, as seen in Figures S5 and S6.

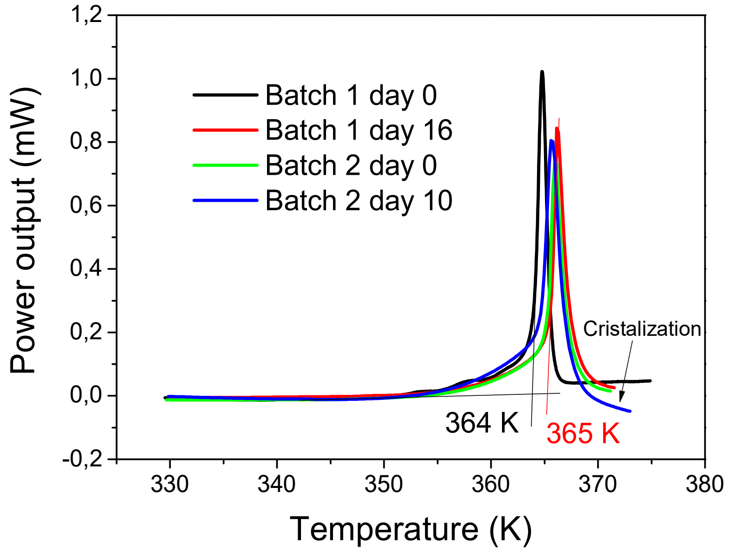

Figure S5. DSC curves of TPD glasses deposited at 285 K = 0.86T_g, in two different evaporations batches and measured at two different moments. The differences in T_{on} arising from temperature or rate inhomogeneities during the evaporation process yield larger variations (1 K) than any possible effect from water absorption during the time elapsed during sample preparation and measurement. An arbitrary straight base line has been subtracted for clear comparison.

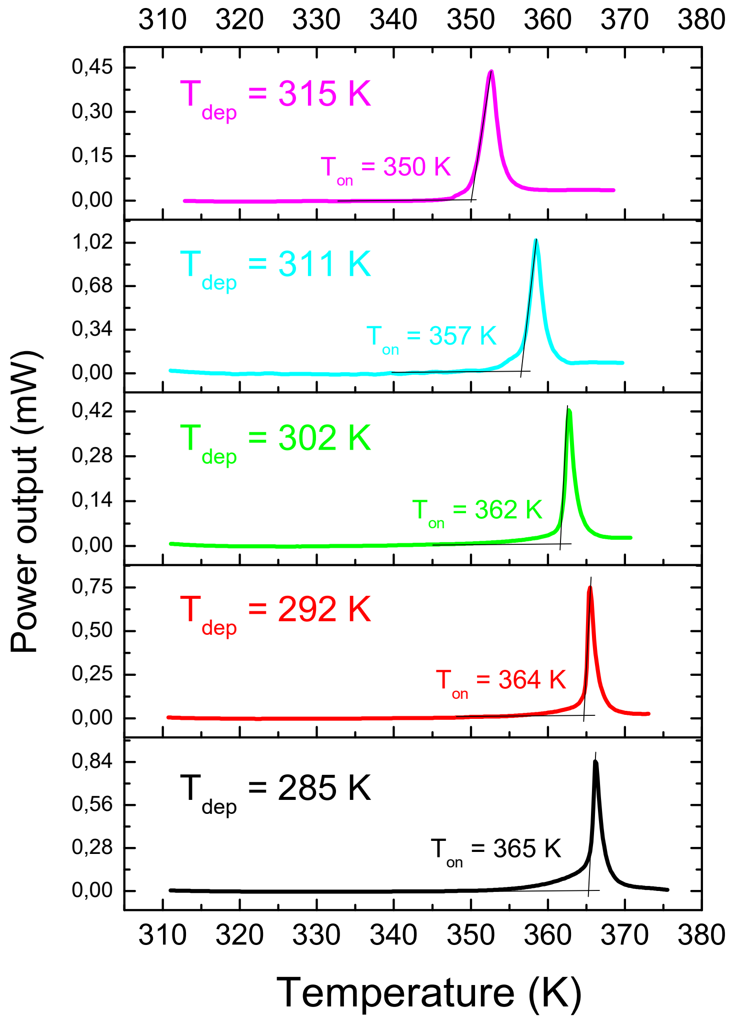

Figure S6. DSC curves of TPD glasses deposited at different substrate temperatures. The aspect of the devitrification peak is not significantly affected by deposition conditions and we do not observe any trace of multiple peak. An arbitrary straight base line has been subtracted for clear comparison.

- Glasses deposited between T_g and around $0.8T_g$ are denser than the ordinary glass produced by cooling from the liquid state. In this deposition temperature range, the closer the temperature to $0.8T_g$, the denser the glass (as manifested also in the previous figure with DSC scans, where lower T_{on} is associated with lower density). As a consequence, these glasses absorb less water than the ones prepared by cooling from the liquid. An example of this was reported for an ultrastable glass of indomethacin, a pharmaceutical material which does absorb water

(*"Highly Stable Indomethacin Glasses Resist Uptake of Water Vapor", J. Phys. Chem. B 2009, 113, 2422–2427*). Therefore, in the unexpected case of water absorption in TPD glasses, we would expect a larger effect in the ordinary glass rather than in the ultrastable glass (deposited at $0.86T_g$, in the case of this work). Since absorbed water increase the conductivity of the sample, its effect would go in an opposite direction as the reported in this work, i.e. glasses deposited closer to T_g, with more absorbed water, would exhibit larger values of conductivity, and the closer the T_{dep} to $0.85T_g$, the lower the conductivity. We observe, however, the opposite result. Furthermore, in our work we also observed that an ordinary glass prepared by cooling from the liquid at ambient conditions does not show any dielectric process in the glassy state.

Furthermore, as observed in the case of indomethacin (*"Highly Stable Indomethacin Glasses Resist Uptake of Water Vapor", J. Phys. Chem. B 2009, 113, 2422–2427*), which is a pharmaceutical compound and susceptible of water absorption, the ordinary glass absorbs around 1% of water (mass %), while the ultrastable absorbs only around 0.1%. In the case of TPD, which is not hydrophilic and not expected to interact with water, we expect much lower values (even negligible, as commented above). In some reported works concerning the effect of absorbed water on the electrical conduction properties of glasses, the samples are intentionally hydrated (several days at ambient conditions) reaching in some cases values of 20% of water in mass%, or even more. For example, in *J. Phys. Chem. C, 2015, 119 (1), pp 685–694*, the authors study the behaviour of hydroscopic materials with water content of around 20%, and report an enhancement of DC conductivity of 2 to 4 orders of magnitude.

As a final remark, we note that it is a general trend that vapour deposited glasses exhibit similar properties, i.e. increased density and slight molecular orientation, which depends on the deposition conditions. If the observed results were strongly affected by water, or if water were key to explain the observed results, then we would expect the same for other vapour deposited glasses, which is not the case. In particular, we do not observe DC conductivity in other vapour deposited glasses as a consequence of any possible water inclusion.
